# Time trends in mortality and life expectancy in 22,658 patients hospitalized with alcohol-associated cirrhosis: A nationwide cohort study

**DOI:** 10.1097/HC9.0000000000000279

**Published:** 2023-09-27

**Authors:** Axel Wester, Ying Shang, Per Stål, Hannes Hagström

**Affiliations:** 1 Department of Medicine, Huddinge, Karolinska Institutet, Stockholm, Sweden; 2 Department of Upper GI, Division of Hepatology, Karolinska University Hospital, Stockholm, Sweden

## Abstract

**Background:**

The management of alcohol-associated cirrhosis has improved in the last decades, but whether the prognosis has changed over time is uncertain. We aimed to assess time trends in mortality and life expectancy in patients hospitalized with alcohol-associated cirrhosis.

**Methods:**

In this population-based cohort study, we used the Swedish national population and health registers to identify all patients with a first episode of in-hospital alcohol-associated cirrhosis from 1969 to 2019 (n = 22,658). Time trends in 1-year mortality were assessed with multivariable Cox regression. A flexible parametric model was fitted to evaluate loss in life expectancy.

**Results:**

Crude mortality was similar in the 2010s and 1980s (unadjusted HR = 1.00, 95% CI = 0.93–1.08, p_trend_ = 0.767). However, when adjusting for baseline characteristics, mortality was lower in the 2010s than in the 1980s (adjusted HR = 0.74, 95% CI = 0.68–0.80), including both liver- and nonliver-related mortalities. These results were consistent in men but not in women, where only nonliver mortality had decreased. The average loss in life expectancy for patients with alcohol-associated cirrhosis compared with the general population was similar throughout the study period (in the 2010s: 14.3 y shorter (95% CI = 13.7–14.9) in men and 15.8 years shorter (95% CI = 14.9–16.7) in women).

**Conclusion:**

Mortality in patients hospitalized with alcohol-associated cirrhosis has improved somewhat when accounting for baseline characteristics, but the loss in life expectancy remains substantial. This underscores the need for new therapeutic options and health policy interventions to further improve the dismal prognosis and life expectancy of patients with alcohol-associated cirrhosis.

## INTRODUCTION

Alcohol-associated liver disease greatly impacts mortality and morbidity worldwide and is the most common cause of liver cirrhosis in many countries, including Sweden.^1^ In addition, alcohol-associated cirrhosis has a worse prognosis than cirrhosis of other causes.^2–4^ Despite improved management of cirrhosis over the last decades, including increased availability of liver transplantation, upper endoscopy to detect and treat varices, TIPS insertion and nonselective beta-blockers to lower portal pressure, and pharmacotherapy, such as terlipressin to treat bleeding varices, the mortality following a diagnosis of alcohol-associated cirrhosis remains substantial, especially for patients in need of hospital care.^5^ However, few studies have examined time trends in mortality in patients with alcohol-associated cirrhosis.^6^

The global mortality burden from alcohol-associated cirrhosis in terms of the total number of deaths is increasing, while the mortality rate in the population per 100,000 inhabitants is decreasing, although this varies between countries.^7–9^ However, whether the mortality has changed for the individual patient following a diagnosis of alcohol-associated cirrhosis is less studied. This was the objective of a Danish cohort study, which found a decreasing trend in all-cause mortality in patients with alcohol-associated cirrhosis diagnosed between 1996 and 2013.^6^ However, the study focused on all-cause mortality, which could disguise a constant or worsening trend in liver-related mortality if the mortality from extrahepatic diseases has improved.^6^ Advances in health care for cirrhosis should primarily translate into lower liver-related mortality. Moreover, whether any changes in mortality have affected the loss in life expectancy of patients with alcohol-associated cirrhosis compared with the general population is unclear. Therefore, the aim of this study was to examine time trends in all-cause, liver-related, and nonliver-related mortalities, as well as loss in life expectancy compared with the general population in patients with a first-time in-hospital diagnosis of alcohol-associated cirrhosis.

## METHODS

### Data sources

The Swedish National Patient Register (NPR) contains data on all International Classification of Disease (ICD) codes from inpatient care since 1964. The positive predictive value of the NPR has been estimated to be 85%–95% in general and specifically 93% for alcohol-associated cirrhosis.^10,11^ This register can be linked to other national registers in Sweden by using the unique personal identification number that is given to all Swedish residents.^12^ The total population register was initiated in 1968 and holds data on country and date of birth, sex, marital status, migration, and death dates.^13^ When a person in Sweden dies, the physician who confirmed the death is obliged to report the death to the Swedish Tax Agency. The coverage of deaths in this register is virtually 100% within 30 days.^13^ Next, the cause of death is reported to the National Board of Health and Welfare within 3 weeks for registration in the Swedish Causes of Death Register, which was established electronically in 1952 and has a completeness above 99%.^14^ In addition, the Human Mortality Database provides open data on mortality rates for the full population of several countries, including Sweden.^15^

### Study population

Previously, we retrieved data from the Swedish national registers on all persons who were diagnosed with any chronic liver disease between 1964 and 2020 in Sweden.^16^ In the current study, we used the NPR to identify all patients of at least 18 years of age who had been diagnosed with alcohol-associated cirrhosis at any hospital in Sweden and discharged alive between January 1, 1969 and December 31, 2019. The definition of alcohol-associated cirrhosis was based on ICD codes (Supplemental Table S1, http://links.lww.com/HC9/A537). The start of follow-up (baseline) was defined as the first hospital discharge with a registered diagnostic code for alcohol-associated cirrhosis during the study period. The end of the inclusion period was December 31, 2019 to allow at least 1 year of follow-up of all cases. Patients were excluded if they had a recorded diagnosis of any chronic liver disease other than alcohol-associated liver disease or had received a liver transplant before or at baseline (Supplemental Table S1, http://links.lww.com/HC9/A537, Supplemental Figure S1, http://links.lww.com/HC9/A537).

### Exposure

The primary exposure was the calendar year of alcohol-associated cirrhosis diagnosis, grouped into decades: 1969–1979, 1980–1989, 1990–1999, 2000–2009, and 2010–2019. The decade 1980–1989 was used as the reference for all analyses. We chose this decade rather than the first because (1) it was during these years that upper endoscopy became available in Sweden to detect varices, meaning that the risk of misclassification bias is higher in the decade before; (2) the NPR became nationwide during this decade; and (3) some of the hospital visits with a diagnosis of alcohol-associated cirrhosis in the first study decade (1969–1979) might not be the patients’ first hospitalization, but rather rehospitalization for patients who survived a first hospitalization before the start of the study, which could introduce selection bias with healthier patients in the first study decade compared with the following decades.

### Subgroups

Subgroup analyses were done based on sex and whether decompensation was present at baseline. Decompensation was defined by ICD coding for ascites, esophageal varices with or without bleeding, hepatorenal syndrome, or liver encephalopathy before or at baseline (Supplemental Table S2, http://links.lww.com/HC9/A537). Esophageal varices without bleeding were included in the definition because the eighth revision of the ICD, which was used before 1987 in Sweden, does not distinguish between bleeding and nonbleeding varices. Only including bleeding varices in the definition for the years after 1987, but not before, would potentially have biased the subgroup with decompensated cirrhosis to be sicker in the later years during the study period.

### Outcomes

The primary outcome was all-cause mortality during the first year after a diagnosis of alcohol-associated cirrhosis. Secondary outcomes included 5-year all-cause mortality and 1- and 5-year liver- and nonliver-related mortalities, based on ICD codes for the main cause of death in the Causes of Death Register (Supplemental Table S3, http://links.lww.com/HC9/A537), 1-year first-time rehospitalization of any cause, and the loss in life expectancy for patients with alcohol-associated cirrhosis compared with the general population.

### Statistics

The number of deaths and the cumulative mortality within 1 and 5 years after the first hospitalization with alcohol-associated cirrhosis were calculated. For cause-specific mortality (ie, liver-related and nonliver-related mortalities), we accounted for competing death causes.^17^ Age-standardized mortality rates were calculated. Two-sided *p* values were estimated to assess trends, considering a *p* value below 0.05 to indicate statistical significance. Moreover, Cox regression models were applied to estimate unadjusted and adjusted HR and their 95% CIs. The multivariable models were adjusted for age, sex, marital status (married or not married), country of birth (Nordic country or other), and the presence at baseline of decompensation or major comorbidities that were selected a priori based on their strong association with mortality: cardiovascular disease, diabetes, dementia, chronic obstructive pulmonary disease, cancer, and chronic kidney disease. Comorbidities were defined by ICD codes (Supplemental Table S2, http://links.lww.com/HC9/A537). In a sensitivity analysis, we additionally adjusted the model for alcohol use disorder diagnosed during follow-up as a proxy for continued heavy drinking. Alcohol use disorder was included as a time-varying covariate and defined according to ICD codes in Supplemental Table S2, http://links.lww.com/HC9/A537. Another sensitivity analysis was done where adjustments were made for age, sex, marital status, country of birth, and a comorbidity index instead of our predefined list of comorbidities. We used a version of the Charlson Comorbidity Index that has been adapted for register-based research in Sweden.^18^ We also did a sensitivity analysis where only bleeding esophageal varices were included in the definition of decompensation. Since we only could distinguish between nonbleeding and bleeding varices from 1987 and onwards, we excluded earlier years from this analysis and used the years 1987–1989 as the reference. In all analyses, patients were followed until death, emigration from Sweden, or until December 31, 2020, whichever occurred first.

The cumulative incidence of first-time rehospitalization of any cause was calculated while accounting for the competing risk of death. Rehospitalization rates were age-standardized, and HRs were estimated using the same models as described above.

In addition, we estimated the loss in life expectancy for patients with alcohol-associated cirrhosis compared with the general population.^19^ Loss in life expectancy was defined as the difference between the mean observed survival time in patients with alcohol-associated cirrhosis and the mean expected survival in the Swedish general population. The Human Mortality Database was used to extract the expected survival rates in the general population, matched to the patients for age, sex, and calendar year of diagnosis.^15^ Flexible parametric models adapted for relative survival (using 6 degrees of freedom for the baseline rate and 4 degrees of freedom for the time-dependent effect) were fitted to estimate the loss in life expectancy as a function of age at diagnosis and stratified by sex.^20,21^ The Akaike information criterion was used to select the degrees of freedom for the model.^20,21^ All analyses were done using Stata 17.0 (StataCorp, College Station, TX, USA). The Regional Ethics Committee of Stockholm approved the study (protocol number 2017/1019-31/1) and waived informed consent.

## RESULTS

### Baseline characteristics

A total of 22,658 patients were diagnosed with alcohol-associated cirrhosis requiring inpatient care at any hospital in Sweden between 1969 and 2019. Comparing the first study decade (1969–1979) to the last (2010–2019), the age at diagnosis increased (median 56 vs. 65 y) and the proportion of women (21.0% vs. 28.1%) (Table 1). Moreover, the proportion of patients born in a Nordic country or who were married at baseline decreased over time, while the education level of the patients was higher in the later study decades. The length of the hospital stay was shorter in recent years compared with earlier. More patients had decompensated cirrhosis and comorbidities in the last study decade compared with the first (Table 1).

**TABLE 1:** Baseline characteristics for patients diagnosed with alcohol-associated cirrhosis in Sweden between 1969 and 2019 (n = 22,658)

	Patients with alcohol-associated cirrhosis					
	1969–1979	1980–1989	1990–1999	2000–2009	2010–2019	Missing data, n (%)
Included persons, n	5677	3821	3517	3930	5713	—
Age at baseline (y), median (IQR)	56 (48–63)	58 (49–65)	60 (51–67)	61 (55–67)	65 (57–70)	0 (0.0)
Sex, n (%)	—	—	—	—	—	0 (0.0)
Men	4485 (79.0)	2867 (75.0)	2618 (74.4)	2862 (72.8)	4108 (71.9)	—
Women	1192 (21.0)	954 (25.0)	899 (25.6)	1068 (27.2)	1605 (28.1)	—
Country of birth, n (%)	—	—	—	—	—	2 (0.0)
Nordic	5557 (97.9)	3707 (97.0)	3354 (95.4)	3718 (94.6)	5340 (93.5)	—
Other	119 (2.1)	113 (3.0)	163 (4.6)	212 (5.4)	373 (6.5)	—
Education >12 y, n (%)	41 (5.1)	110 (8.3)	306 (10.0)	563 (14.5)	1096 (19.3)	7,892 (34.8)
Married, n (%)	2557 (45.1)	1469 (38.5)	1345 (38.2)	1497 (38.1)	2124 (37.2)	1 (0.0)
Length of hospital stay (d), median (IQR)	14 (7–26)	12 (6–22)	8 (4–15)	7 (3–12)	5 (2–10)	1 (0.0)
Decompensation, n (%)	710 (12.5)	894 (23.4)	1225 (34.8)	1856 (47.2)	2795 (48.9)	0 (0.0)
Comorbidity before or at baseline, n (%)	—	—	—	—	—	—
Cardiovascular disease	810 (14.3)	687 (18.0)	849 (24.1)	1339 (34.1)	2711 (47.5)	0 (0.0)
Diabetes	609 (10.7)	447 (11.7)	497 (14.1)	694 (17.7)	1298 (22.7)	0 (0.0)
Dementia	32 (0.6)	20 (0.5)	28 (0.8)	55 (1.4)	97 (1.7)	0 (0.0)
Chronic obstructive pulmonary disease	134 (2.4)	123 (3.2)	125 (3.6)	240 (6.1)	509 (8.9)	0 (0.0)
Cancer	—	—	—	—	—	0 (0.0)
HCC	24 (0.4)	23 (0.6)	21 (0.6)	51 (1.3)	170 (3.0)	—
Other cancer	154 (2.7)	165 (4.3)	246 (7.0)	334 (8.5)	499 (8.7)	—
Chronic kidney disease	23 (0.4)	15 (0.4)	16 (0.5)	49 (1.3)	190 (3.3)	0 (0.0)
Alcohol use disorder	1923 (33.9)	1865 (48.8)	1728 (49.1)	2115 (53.8)	2985 (52.3)	0 (0.0)

Abbreviation: IQR, interquartile range.

### Mortality trends in the whole study population

**FIGURE 1:**
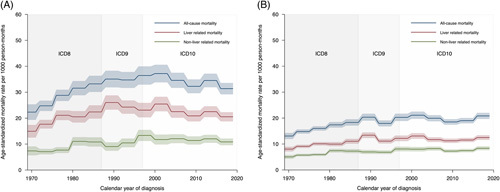
Time trends in mortality per 1000 person-months during the first 1 (A) and 5 (B) years after a diagnosis of alcohol-associated cirrhosis in Sweden between 1969 and 2019. The shaded areas represent 95% CI.

During 1980–1989, the all-cause mortality in patients diagnosed with alcohol-associated cirrhosis at the hospital was 30.5% (95% CI = 29.0–31.9) within 1 year of hospital discharge compared with 30.4% (95% CI = 29.3–31.6) during 2010–2019 (Table 2). Likewise, the age-standardized mortality rate (32.7 per 1000 person-months, 95% CI = 30.8–34.5 vs. 32.7 per 1000 person-months, 95% CI = 31.1–34.2) was stable across time after the 1980s (unadjusted HR 1.00, 95% CI = 0.93–1.08, p_trend_ = 0.767) (Table 2, Figure 1A). The median survival was 3.1 years in both the 1980s and in the 2010s (Supplemental Table S4, http://links.lww.com/HC9/A537). However, when adjusting for baseline characteristics, the mortality rate was found to have declined over time with an adjusted HR in the last decade of 0.74 (95% CI = 0.68–0.80) compared with that during 1980–1989 (Table 2). Similar results were found when analyzing liver-related and nonliver-related mortalities separately (Table 2, Figure 1A), for 5-year mortality (Table 3, Figure 1B), and when adjusting for alcohol use disorder during follow-up as a proxy for continued heavy drinking (Supplemental Table S5, http://links.lww.com/HC9/A537). Results were also similar when adjusting for a comorbidity index instead of separate comorbidity variables (Supplemental Table S6, http://links.lww.com/HC9/A537).

**TABLE 2:** One-year mortality in patients with alcohol-associated cirrhosis (n = 22,658)

	Number of deaths	Cumulative 1-year mortality (%), (95% CI)	Age-standardized 1-year mortality rate per 1000 person-months, (95% CI)	*p* value for trend^a^	Unadjusted HR (95% CI)	Adjusted HR^b^ (95% CI)
All-cause	—	—	—	0.767	—	—
1969–1979	1461	25.7 (24.6–26.9)	26.0 (24.7–27.3)	—	0.81 (0.75–0.88)	0.90 (0.83–0.97)
1980–1989	1164	30.5 (29.0–31.9)	32.7 (30.8–34.5)	—	Reference	Reference
1990–1999	1135	32.3 (30.7–33.8)	35.6 (33.5–37.7)	—	1.07 (0.99–1.16)	0.98 (0.90–0.94)
2000–2009	1258	32.0 (30.6–33.5)	34.9 (33.0–36.9)	—	1.06 (0.98–1.15)	0.87 (0.80–0.94)
2010–2019	1738	30.4 (29.3–31.6)	32.7 (31.1–34.2)	—	1.00 (0.93–1.08)	0.74 (0.68–0.80)
Liver-related	—	—	—	0.118	—	—
1969–1979	1050	18.5 (17.5–19.5)	18.6 (17.5–19.7)	—	0.85 (0.78–0.93)	0.94 (0.86–1.03)
1980–1989	800	20.9 (19.7–22.3)	22.2 (20.7–23.8)	—	Reference	Reference
1990–1999	773	22.0 (20.6–23.4)	24.2 (22.4–25.9)	—	1.06 (0.96–1.17)	0.97 (0.88–1.07)
2000–2009	845	21.5 (20.2–22.8)	23.4 (21.8–25.0)	—	1.04 (0.94–1.14)	0.87 (0.78–0.96)
2010–2019	1127	19.7 (18.7–20.8)	21.1 (19.9–22.3)	—	0.95 (0.86–1.03)	0.76 (0.69–0.83)
Nonliver-related	—	—	—	0.087	—	—
1969–1979	411	7.2 (6.6–7.9)	7.4 (6.7–8.1)	—	0.73 (0.63–0.84)	0.81 (0.70–0.93)
1980–1989	364	9.5 (8.6–10.5)	10.4 (9.3–11.5)	—	Reference	Reference
1990–1999	362	10.3 (9.6–11.5)	11.4 (10.2–12.6)	—	1.10 (0.95–1.27)	0.98 (0.85–1.14)
2000–2009	413	10.5 (9.6–11.5)	11.5 (10.4–12.6)	—	1.12 (0.97–1.28)	0.87 (0.76–1.01)
2010–2019	611	10.7 (9.9–11.5)	11.6 (10.6–12.5)	—	1.13 (0.99–1.28)	0.70 (0.61–0.81)

a The decade 1980–1989 was used as a reference.

b Adjusted for age, sex, marital status, country of birth, decompensation, cardiovascular disease, diabetes, dementia, chronic obstructive pulmonary disease, cancer, and chronic kidney disease.

**TABLE 3:** Five-year mortality in patients with alcohol-associated cirrhosis (n = 22,658)

	Number of deaths	Cumulative 5-year mortality (%), (95% CI)	Age-standardized 5-year mortality rate per 1000 person-months, (95% CI)	*p* value for trend^a^	Unadjusted HR (95% CI)	Adjusted HR^b^ (95% CI)
All-cause	—	—	—	0.706	—	—
1969–1979	3113	54.9 (53.6–56.2)	15.0 (14.5–15.5)	—	0.85 (0.81–0.90)	0.92 (0.87–0.97)
1980–1989	2305	60.5 (58.9–62.0)	18.1 (17.4–18.9)	—	Reference	Reference
1990–1999	2172	61.8 (60.2–63.4)	19.4 (18.5–20.2)	—	1.05 (0.99–1.11)	0.97 (0.91–1.03)
2000–2009	2467	63.0 (61.4–64.5)	19.8 (19.0–20.6)	—	1.07 (1.01–1.14)	0.90 (0.85–0.96)
2010–2019	3119	61.1 (59.7–62.5)	19.8 (19.1–20.5)	—	1.01 (0.96–1.07)	0.77 (0.73–0.82)
Liver-related	—	—	—	0.553	—	—
1969–1979	1954	34.5 (33.2–35.7)	9.3 (8.9–9.7)	—	0.88 (0.82–0.94)	0.95 (0.89 to 1.02)
1980–1989	1403	36.8 (35.3–38.3)	10.9 (10.3–11.4)	—	Reference	Reference
1990–1999	1359	38.7 (37.1–40.3)	12.0 (11.4–12.7)	—	1.07 (1.00–1.16)	1.00 (0.92–1.07)
2000–2009	1506	38.4 (36.9–39.9)	12.0 (11.4–12.6)	—	1.07 (1.00–1.15)	0.92 (0.86–0.99)
2010–2019	1889	36.6 (35.2–38.0)	11.9 (11.3–12.4)	—	0.99 (0.92–1.06)	0.82 (0.76–0.88)
Nonliver-related	—	—	—	0.176	—	—
1969–1979	1159	20.5 (19.4–21.5)	5.7 (5.3–6.0)	—	0.81 (0.74–0.88)	0.87 (0.80–0.95)
1980–1989	902	23.7 (22.3–25.0)	7.3 (6.8–7.7)	—	Reference	Reference
1990–1999	813	23.2 (21.8–24.6)	7.3 (6.8–7.8)	—	1.00 (0.91–1.10)	0.93 (0.84–1.02)
2000–2009	961	24.5 (23.2–25.9)	7.8 (7.3–8.3)	—	1.07 (0.98–1.18)	0.87 (0.80–0.96)
2010–2019	1230	24.5 (23.3–25.7)	7.9 (7.4–8.3)	—	1.05 (0.96–1.14)	0.70 (0.64–0.77)

a The decade 1980–1989 was used as a reference.

b Adjusted for age, sex, marital status, country of birth, decompensation, cardiovascular disease, diabetes, dementia, chronic obstructive pulmonary disease, cancer, and chronic kidney disease.

### Mortality trends in subgroups

**FIGURE 2:**
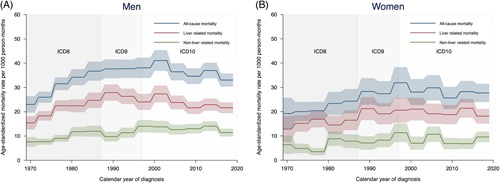
Time trends in mortality per 1000 person-months during the first year after a diagnosis of alcohol-associated cirrhosis in Sweden between 1969 and 2019 for men (A) and women (B). The shaded areas represent 95% CI.

Men with alcohol-associated cirrhosis (n = 16,940, 74.8%) had an unchanging all-cause mortality after the 1980s, contrasted by a falling liver-related mortality (p_trend_ = 0.023) and a rising nonliver-related mortality (p_trend_ = 0.039) (Supplemental Table S7, http://links.lww.com/HC9/A537, Figure 2A). The adjusted estimates showed declining mortality regardless of the cause of death (Supplemental Table S7, http://links.lww.com/HC9/A537). Women (n = 5718, 25.2%), on the other hand, did not show any statistically significant time trends in mortality (Supplemental Table S8, http://links.lww.com/HC9/A537, Figure 2B). These results remained for all-cause and liver-related mortality when adjustments were made for baseline characteristics but changed for nonliver-related mortality, which was found to be lower in the last decade compared with the 1980s (adjusted HR 0.68, 95% CI = 0.49-0.93) (Supplemental Table S8, http://links.lww.com/HC9/A537).

Patients with compensated alcohol-associated cirrhosis (n = 15,178, 67.0%) had similarly high all-cause mortality in the 2010s as in the 1980s (HR 1.00, 95% CI = 0.91–1.10, p_trend_ = 0.785) (Supplemental Table S9, http://links.lww.com/HC9/A537). There was a trend of decreasing liver-related mortality (HR 0.88, 95% CI = 0.77–0.99, p_trend_ = 0.033), paralleled by an increasing nonliver-related mortality (HR 1.22, 95% CI = 1.04–1.42, p_trend_ = 0.026) (Supplemental Table S9, http://links.lww.com/HC9/A537, Supplemental Figure S2A, http://links.lww.com/HC9/A537). However, the multivariable analysis showed that the mortality rate from any cause had decreased over time when the baseline characteristics were balanced between the groups (Supplemental Table S9, http://links.lww.com/HC9/A537). In contrast to patients with compensated cirrhosis, patients with decompensation at baseline (n = 7480, 33.0%) had a trend of declining all-cause mortality over time (HR 0.87, 95% CI = 0.77–0.99, p_trend_ = 0.014), with 1-year estimates of 36.2% in the 1980s compared with 32.4% in the 2010s (Supplemental Table S10, http://links.lww.com/HC9/A537, Supplemental Figure S2B, http://links.lww.com/HC9/A537). This finding was driven by a decreasing liver-related mortality (HR 0.80, 95% CI = 0.69–0.92, p_trend_<0.001), whereas the nonliver-related mortality was stable (HR 1.17, 95% CI = 0.89–1.53, p_trend_ = 0.108). Here, results were consistent after adjusting for baseline characteristics (Supplemental Table S10, http://links.lww.com/HC9/A537). These analyses of patients with compensated and decompensated cirrhosis generated similar results when only bleeding varices were included in the definition of decompensation (Supplemental Table S11, http://links.lww.com/HC9/A537, Supplemental Table S12, http://links.lww.com/HC9/A537).

### Rehospitalization trends

Rehospitalization estimates are presented in Supplemental Table S13, http://links.lww.com/HC9/A537. There was a trend of increasing crude rehospitalization rates in the 2010s compared with the 1980s (unadjusted HR 1.07, 95% CI = 1.02–1.12, p_trend_ = 0.004), whereas the adjusted rates were stable (HR 0.96, 95% CI = 0.91–1.01). In the 2010s, 73.8% of the patients were rehospitalized within 1 year after their first in-hospital diagnosis of alcohol-associated cirrhosis.

### Loss in life expectancy

**FIGURE 3:**
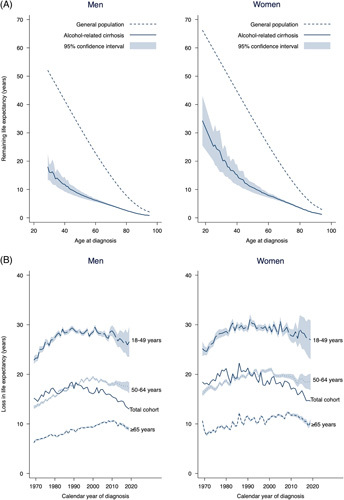
Remaining life expectancy in patients with alcohol-associated cirrhosis and the general population during 2010–2019 (A) and changes in the loss in life expectancy between 1969 and 2019, stratified by age group at first diagnosis (B).

The average loss in life expectancy in patients of any age with alcohol-associated cirrhosis compared with the general population dropped during the study period (Table 4). In the 1980s, men with alcohol-associated cirrhosis had an average of 17.0 years of lost life compared with 14.3 years during 2010–2019. The corresponding numbers for women were 20.2 and 15.8 years. Alcohol-associated cirrhosis was associated with a loss in life expectancy regardless of the age at diagnosis, with particularly large losses for younger patients (Figure 3A). Since the age distribution of our study population changed over time, we also analyzed the loss in life expectancy within different age groups (18–49, 50–64, and ≥65 y). In contrast to the analysis of patients of any age, this analysis showed that the loss in life expectancy has remained stable within all these age groups over the last decades (Figure 3B).

**TABLE 4:** Average loss in life expectancy in patients of any age with alcohol-associated cirrhosis (n = 22,658) compared to the general population

	Average loss in life expectancy (y), (95% CI)	
Inclusion period	Men	Women
1969–1979	16.0 (15.7–16.3)	19.3 (18.8–19.8)
1980–1989	17.0 (16.7–17.2)	20.2 (19.8–20.6)
1990–1999	17.3 (17.1–17.6)	19.4 (19.0–19.7)
2000–2009	16.4 (16.1–16.6)	18.4 (18.0–18.8)
2010–2019	14.3 (13.7–14.9)	15.8 (14.9–16.7)

## DISCUSSION

The main findings of this nationwide population-based study of time trends in mortality and life expectancy following a first-time hospitalization for alcohol-associated cirrhosis are given as follows. First, patients had more comorbidities and were older at diagnosis in the later study decades. Second, the crude mortality in the whole study population was stable over time. Third, when baseline characteristics were balanced, there was a decreasing trend in mortality for the whole study population and across all subgroups except women. Fourth, the loss in life expectancy for patients with alcohol-associated cirrhosis remains substantial. On average, a patient with alcohol-associated cirrhosis can today expect at least 14–16 years shorter life span than the general population.

Liver cirrhosis constitutes a great clinical problem with a dismal prognosis for the affected patient.^2^ Studies indicate that the in-hospital mortality in patients hospitalized with cirrhosis has decreased but shifted to the immediate post-discharge period.^22^ We studied mortality during the first 1 and 5 years after a first-time hospitalization for alcohol-associated cirrhosis in Sweden and found the overall trend to be stagnant, although there was an increase in the 1990s followed by a decline in the most recent decade. This finding supplements a previous study of first-time hospitalizations for patients with alcohol-associated liver disease in Sweden (with or without cirrhosis) that found an increase in mortality between 1969 and 2006.^23^ In addition, the stagnant trend in crude mortality is in line with a prior study from Sweden on patients with biopsy-proven alcohol-associated liver disease, where mortality rates compared with the general population were similar from the 1980s and forward.^24^ More specifically, we showed that the trend in mortality was, to a high degree, caused by changes in patient age and comorbidity status, as the mortality was found to be decreasing when accounting for these factors. This agrees with a Danish cohort study that also found decreasing mortality for patients with alcohol-associated cirrhosis when accounting for differences in baseline characteristics over time.^6^ Our study adds to the evidence from the Danish study by further examining cause-specific mortality.^6^ We found that, when accounting for baseline characteristics, both the liver-related and nonliver-related mortalities had decreased over time.

We further compared men and women and found that, when accounting for differences in baseline characteristics across time, only men had a declining trend in all-cause mortality, while the mortality in women was unchanged. The finding in women might be explained by a lack of statistical power in the smaller women cohort or by sex-related inequality regarding access to specialized health care. For example, a previous study of patients with cirrhosis found that men were more likely than women to receive a liver transplant despite having similar age, Child-Pugh score, etiology of cirrhosis, and frequency of diabetes.^3^

Patients with compensated cirrhosis were found to have declining liver-related but increasing nonliver-related mortality, resulting in an unchanging all-cause mortality over time. Adjustments for baseline characteristics did, however, reveal a decrease in mortality regardless of the cause. Moreover, patients with decompensated cirrhosis had a trend of decreasing all-cause mortality driven by falling liver-related mortality, which is in line with a prior study that showed improved prognosis for patients hospitalized with esophageal varices in Sweden.^25^

The improved prognosis that we observed when accounting for differences in baseline characteristics is reflected by a decreasing average loss in life expectancy over time in patients of any age with alcohol-associated cirrhosis compared with the general population in our study. However, despite this trend, patients with alcohol-associated cirrhosis still have considerably lower life expectancy than the general population (14.3 y lower for men and 15.8 y for women in the 2010s). These results concur with a previous study by Westman and colleagues that found a 24–28 years shorter life expectancy for patients with alcohol use disorder in the Nordic countries compared with the general population.^26^ The greater loss in that study compared with ours is probably due to differences in the age at diagnosis between alcohol use disorder and alcohol-associated cirrhosis. In addition, Westman et al found that the loss in life expectancy increased over time for men but decreased for women.^26^ We found a decreasing trend in the loss in life expectancy for both men and women when analyzing patients of any age. However, when looking at changes in specific age groups, the loss in life expectancy was found to have been constant during the last 30 years. A plausible explanation for this finding would be that the age distribution of our cohort shifted toward the higher ages in later decades, reflecting an older age at presentation, and older patients generally have a smaller loss in life expectancy compared with patients who are diagnosed at a younger age.

We observed differences in baseline characteristics over the study period. For instance, patients were diagnosed at an older age, were more frequently decompensated, and had more comorbidities such as diabetes and cancer in the later study decades. These changes are likely highly associated with one another and explained by several factors. Sweden has seen a decline in the number of available hospital beds, which is also reflected by our finding of a reduction in the length of hospital stay over time. Hence, admission to the wards has become more restrictive, resulting in older and sicker patients with more comorbidities in the hospitals. The increase in comorbidities is likely primarily explained by an increase in age at first hospitalization with alcohol-associated cirrhosis. Moreover, the observed rise in the proportion of patients with decompensation at the time of hospitalization may partly be explained by the advent of upper endoscopy to detect varices, which were probably underreported in the first study decades. There might also be differences in the incidence of alcohol-associated cirrhosis between birth cohorts due to changing patterns of alcohol consumption, which could result in cirrhosis development earlier in life at the beginning of our study period and, thereby, lower age at diagnosis.^27^ We saw a great increase in our cohort size in the last study decade. This could indicate a true increase in the incidence of alcohol-associated cirrhosis, increased detection of cirrhosis with the gradual introduction of modern noninvasive techniques to diagnose cirrhosis, or a combination of both.

### Clinical and health policy implications

Although the prognosis following hospitalization for alcohol-associated cirrhosis seems to have improved, mortality remains high, with estimates for cumulative mortality at 30% within 1 year of hospital discharge and 61% within 5 years, twice that of the 1- and 5-year estimates following a diagnosis of myocardial infarction or any cancer, respectively.^28,29^ In addition, the incidence of alcohol-associated cirrhosis might be increasing, as indicated by previous work and the almost 50% increase in the number of patients hospitalized in the 2010s compared with the 2000s in our study.^6^ These results underscore the need for new treatment options to improve the prognosis of cirrhosis further, as well as earlier detection of precirrhotic liver disease. In addition, the prognosis of alcohol-associated cirrhosis has considerable implications for public health. Chronic liver disease is the second leading cause of years of working life lost and caused nearly 300,000 premature deaths per year in Europe.^30^ Decreases have been observed for the global mortality rate from alcohol-associated cirrhosis, although this trend varies between countries.^7–9^ As shown by us and others, alcohol-associated cirrhosis is associated with a substantial number of years of lost life.^31^ Importantly, global alcohol consumption has increased in the last decades and is expected to increase further in the future, resulting in a potentially higher mortality burden from alcohol-associated liver disease unless preventive interventions are implemented.^31,32^

### Strengths and limitations

The main strength of our study is the use of the validated Swedish population-based registers to ascertain our outcomes and include a large sample of study participants across half a century on a national level, minimizing selection bias and rendering high precision and accuracy.^10,11,13,14^ Some limitations should be noted when interpreting our results. First, we did not have data on Child-Pugh or the Model for End-Stage Liver Disease scores to further delineate the severity of cirrhosis beyond the presence or absence of decompensation. Second, 3 different versions of the ICD system were used during the study period. Validation studies of the Swedish registers have mainly been made for the tenth revision of the ICD system, although available data indicate similarly high validity for historical versions.^11^ Third, it is not possible from our results to ascertain the specific reasons for the trends in mortality. Advanced management of cirrhosis-specific complications has likely led to a better prognosis, including the management of bleeding esophageal varices.^25,33^ The observed improvements in both liver-related and nonliver-related mortality rates that we observed in our fully adjusted models do, however, suggest enhanced overall care of patients with cirrhosis. Moreover, many other factors changed during our long study period that may have impacted our results. There have been changes in socioeconomic factors such as education and changes in the amount and type of alcohol consumed in Sweden. For example, the consumption of alcohol increased when Sweden joined the European Union in 1995 due to increased availability and lower prices.^34^ Notwithstanding the causes, our study indicates that the prognosis of alcohol-associated cirrhosis remains poor and associated with a considerably shorter life span than the general population.

## CONCLUSIONS

The trend in mortality after a first-time hospitalization for alcohol-associated cirrhosis in Sweden has been stagnant since the 1980s. However, accounting for differences in baseline characteristics over time, we found a decline in all-cause, liver-related, and nonliver-related mortalities. Despite improved survival in patients with alcohol-associated cirrhosis, the average life expectancy is still 14–16 years shorter than the general population. Our results highlight the need for new therapeutic options and health policy interventions to further improve the dismal prognosis and life expectancy of patients with alcohol-associated cirrhosis.

## Supplementary Material

## DATA SHARING

No data are available due to Swedish regulations.
