# Supplementary material for: Time trends in mortality and life expectancy in 22,658 patients hospitalized with alcohol-associated cirrhosis: A nationwide cohort study
Source: Hepatol Commun. 2023 Sep 27;7(10):e0279. doi: 10.1097/HC9.0000000000000279 (PMC10531483; doi:10.1097/HC9.0000000000000279)
Supplement: Supplementary file 1 [file hc9-7-e0279-s001.pdf]

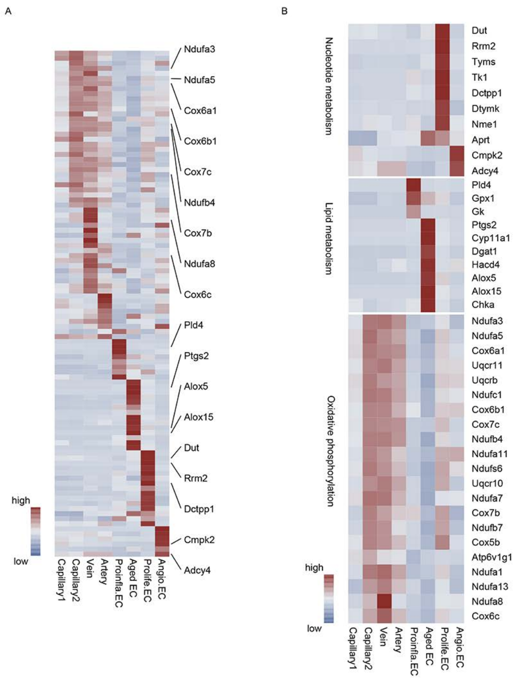
A
Ndufa3
Ndufa5
Cox6a1
Cox6b1
Cox7c
Ndufb4
Cox7b
Ndufa8
Cox6c
Pld4
Ptgs2
Alox5
Alox15
Dut
Rrm2
Dctpp1
Cmpk2
Adcy4
high
low
Capillary1
Capillary2
Vein
Artery
Proinfla.EC
Aged EC
Prolife.EC
Angio.EC
B
Nucleotide metabolism
Dut
Rrm2
Tyms
Tk1
Dctpp1
Dtymk
Nme1
Aprt
Cmpk2
Adcy4
Lipid metabolism
Pld4
Gpx1
Gk
Ptgs2
Cyp11a1
Dgat1
Hacd4
Alox5
Alox15
Chka
Oxidative phosphorylation
Ndufa3
Ndufa5
Cox6a1
Uqcr11
Uqcrb
Ndufc1
Cox6b1
Cox7c
Ndufb4
Ndufa11
Ndufs6
Uqcr10
Ndufa7
Cox7b
Ndufb7
Cox5b
Atp6v1g1
Ndufa1
Ndufa13
Ndufa8
Cox6c
high
low
Capillary1
Capillary2
Vein
Artery
Proinfla.EC
Aged EC
Prolife.EC
Angio.EC

Figure S2

Figure S2. The metabolic heterogeneity of different ECs.

(A) Heatmap showing the mean expression of the metabolic genes in different ECs. Color scale: red, high expression; blue, low expression.

(B) Vascular-Bed specific EC expression heatmaps of representative metabolic genes involved in Nucleotide metabolism, lipid metabolism, and Oxidative phosphorylation metabolism. Color scale: red, high expression; blue, low expression.
