# Supplementary material for: Time trends in mortality and life expectancy in 22,658 patients hospitalized with alcohol-associated cirrhosis: A nationwide cohort study
Source: Hepatol Commun. 2023 Sep 27;7(10):e0279. doi: 10.1097/HC9.0000000000000279 (PMC10531483; doi:10.1097/HC9.0000000000000279)
Supplement: Supplementary file 2 [file hc9-7-e0279-s002.docx]

Supplemental material

Time trends in mortality and life expectancy in 22,658 patients hospitalized with alcohol-related cirrhosis: A nationwide cohort study

Axel Wester, Ying Shang, Per Stål, Hannes Hagström

Table of contents

Page 4 **Supplemental Table 1.** International Classification of Disease (ICD) codes for alcohol-related cirrhosis, other liver diseases, and liver transplantation.

Page 5 **Supplemental Table 2**. International Classification of Disease (ICD) codes for decompensation, comorbidities, and alcohol use disorder.

Page 6 **Supplemental Table 3**. International Classification of Disease (ICD) codes for causes of death.

Page 7 **Supplemental Table 4.** Median survival in patients with alcohol-related cirrhosis (n=22,658).

Page 8 **Supplemental Table 5.** Sensitivity analysis of one-year all-cause mortality in patients with alcohol-related cirrhosis (n=22,658) including alcohol use disorder during follow-up as a time-varying covariate in the model.

Page 9 **Supplemental Table 6.** Sensitivity analysis of one-year mortality in patients with alcohol-related cirrhosis (n=22,658). Adjustments are made for a comorbidity index instead of individual comorbidities.

Page 10 **Supplemental Table 7.** One-year mortality in men with alcohol-related cirrhosis (n=16,940).

Page 11 **Supplemental Table 8.** One-year mortality in women with alcohol-related cirrhosis (n=5718).

Page 12 **Supplemental Table 9.** One-year mortality in patients with compensated alcohol-related cirrhosis including non-bleeding oesophageal varices between 1987 and 2019 (n=8964).

Page 13 **Supplemental Table 10.** One-year mortality in patients with decompensated alcohol-related cirrhosis excluding non-bleeding oesophageal varices between 1987 and 2019 (n=5268).

Page 14 **Supplemental Table 11.** Sensitivity analysis of one-year mortality in patients with compensated alcohol-related cirrhosis between 1987 and 2019 including non-bleeding oesophageal varices (n=8964).

Page 15 **Supplemental Table 12.** Sensitivity analysis of one-year mortality in patients with decompensated alcohol-related cirrhosis between 1987 and 2019 excluding non-bleeding oesophageal varices (n=5268).

Page 16 **Supplemental Table 13.** One-year first-time rehospitalization rates in patients with alcohol-related cirrhosis (n=22,658).

Page 17 **Supplemental Figure 1.** Flowchart of the study population.

Page 18 **Supplemental Figure 2.** Time trends in mortality per 1000 person-months during the first year after a diagnosis of alcohol-related cirrhosis in Sweden between 1969 and 2019 for patients with compensated cirrhosis (A), and decompensated cirrhosis (B). The shaded areas represent the 95% confidence intervals.

**Supplemental Table 1.** International Classification of Disease (ICD) codes for alcohol-related cirrhosis, other liver diseases, and liver transplantation.

|  | **ICD10** | **ICD9** | **ICD8** |
| --- | --- | --- | --- |
| Alcohol-related cirrhosis | K70.3 | 571C | 571.00 |
| Viral hepatitis | B16, B17, B18, B19 | 070, 571E | 070, 999.2 |
| Primary biliary cholangitis | K74.3, K74.5 | 571G | - |
| Primary sclerosing cholangitis | (K50 or K51) +K83.0 | (555 or 556) + 576B | 563+575.05 |
| Autoimmune hepatitis | K75.4 | - | - |
| Budd-Chiari syndrome | I82.0, K76.5 | 453A | - |
| Non-alcoholic fatty liver disease | K76.0, K75.8 | 571W | - |
| Alpha-1 antitrypsin-deficiency | E88.0A, E88.0B | 277G | - |
| Wilson’s disease | E83.0B | 275B | 273.30 |
| Hemochromatosis | E83.1 | 275A | 273.20 |
| Liver cirrhosis, unspecified | K74.6 | 571F | 571.9 |
| Liver transplantation |  |  |  |
| Diagnostic codes | Z94.4 | V42H | - |
| Procedure codes | JJC00, JJC10, JJC20, DJ005, DJ006, JJC30, JJC40 | 5200 | 5200 |

**Supplemental Table 2**. International Classification of Disease (ICD) codes for decompensation, comorbidities, and alcohol use disorder.

|  | **ICD10** | **ICD9** | **ICD8** |
| --- | --- | --- | --- |
| **Decompensation** |  |  |  |
| Ascites | R18.9 | 789F | 785.3 |
| Oesophageal varices with or without bleeding | I85.0, I98.3, I85.9, I98.2 | 456A, 456B, 456C | 456.0 |
| Hepatorenal syndrome | K76.7 | 572E | - |
| Liver encephalopathy | - | 572C | 573.02 |
| **Comorbidities** |  |  |  |
| Cardiovascular disease | Ischemic heart disease: I20-I25  Cerebrovascular disease: I60-I69  Heart failure: I50  Hypertension: I10-I15  Hyperlipidemia: E78  Arterial thrombosis: I74  Peripheral artery disease: I73.9 | Ischemic heart disease: 410-414  Cerebrovascular disease: 430-438  Heart failure: 428  Hypertension: 401-405  Hyperlipidemia: 272A-E  Arterial thrombosis: 444  Peripheral artery disease: 443X | Ischemic heart disease: 410-414  Cerebrovascular disease: 430-438  Heart failure: 427.0/1, 428.9  Hypertension: 400-404  Hyperlipidemia: 272.0/1  Arterial thrombosis: 444  Peripheral artery disease: 443.90 |
| Diabetes | E10, E11 | 250 | 250 |
| Dementia | F00-F03, F051, F107A, G30 | 290, 294B, 331A | 290.0/1 |
| Chronic obstructive pulmonary disease | J41-J44 + age≥40 | 491-492, 496 + age≥40 | 490-492 + age≥40 |
| Cancer |  |  |  |
| Hepatocellular carcinoma | C220 | 155A | 155.01 |
| Other cancers | C00-C97 (except C220) | 140-208 (except 155A) | 140-209 (except 155.01) |
| Chronic kidney disease | I131, I132, I120, N18 | 585 | 582, 792.99 |
| **Alcohol use disorder** |  |  |  |
|  | F10, X65, Y15, Y90, Y91, R78 | 303, 305A, 790D | 303 |

**Supplemental Table 3**. International Classification of Disease (ICD) codes for causes of death.

|  | **ICD10** | **ICD9** | **ICD8** |
| --- | --- | --- | --- |
| **Liver related mortality** |  |  |  |
| Liver disease, all | K70-K77 | 571-573 | 570-573 |
| Oesophageal varices | I85.0, I98.3, I85.9, I98.2 | 4560, 4561, 4562 | 456.0 |
| Hepatocellular carcinoma | C220 | 1550 | 155.01 |
| **Non-liver related mortality** | Any code not corresponding to the above definitions | Any code not corresponding to the above definitions | Any code not corresponding to the above definitions |

**Supplemental Table 4.** Median survival in patients with alcohol-related cirrhosis (n=22,658).

|  | **Median survival (years), (95% CI)** |
| --- | --- |
| 1969-1979 | 4.0 (3.8-4.3) |
| 1980-1989 | 3.1 (2.9-3.4) |
| 1990-1999 | 2.9 (2.6-3.2) |
| 2000-2009 | 2.7 (2.5-2.9) |
| 2010-2019 | 3.1 (2.9-3.3) |

§ Abbreviations: CI, confidence interval**Supplemental Table 5.** Sensitivity analysis of one-year all-cause mortality in patients with alcohol-related cirrhosis (n=22,658) including alcohol use disorder during follow-up as a time-varying covariate in the model.

|  | **Adjusted HR†**  **(95% CI)‡** |
| --- | --- |
| **All-cause** |  |
| 1969-1979 | 0.91 (0.84 to 0.98) |
| 1980-1989 | Reference |
| 1990-1999 | 0.98 (0.90 to 1.06) |
| 2000-2009 | 0.86 (0.80 to 0.94) |
| 2010-2019 | 0.73 (0.67 to 0.79) |

†Adjusted for age, marital status, country of birth, decompensation, cardiovascular disease, diabetes, dementia, chronic obstructive pulmonary disease, cancer, and chronic kidney disease at baseline as well as alcohol use disorder as a time-varying covariate.

‡ Abbreviations: CI, confidence interval; HR, hazard ratio

**Supplemental Table 6.** Sensitivity analysis of one-year mortality in patients with alcohol-related cirrhosis (n=22,658). Adjustments are made for a comorbidity index instead of individual comorbidities.

|  | **Adjusted HR†**  **(95% CI)‡** |
| --- | --- |
| **All-cause** |  |
| 1969-1979 | 0.90 (0.83-0.97) |
| 1980-1989 | Reference |
| 1990-1999 | 0.96 (0.89-1.04) |
| 2000-2009 | 0.87 (0.80-0.94) |
| 2010-2019 | 0.74 (0.69-0.80) |
| **Liver related** |  |
| 1969-1979 | 0.91 (0.83-1.00) |
| 1980-1989 | Reference |
| 1990-1999 | 0.99 (0.89-1.09) |
| 2000-2009 | 0.90 (0.81-0.99) |
| 2010-2019 | 0.76 (0.69-0.84) |
| **Non-liver related** |  |
| 1969-1979 | 0.87 (0.75-1.00) |
| 1980-1989 | Reference |
| 1990-1999 | 0.92 (0.79-1.06) |
| 2000-2009 | 0.82 (0.71-0.95) |
| 2010-2019 | 0.70 (0.62-0.81) |

†Adjusted for age, sex, marital status, country of birth, and an adapted version of the Charlson comorbidity index.

‡ Abbreviations: CI, confidence interval; HR, hazard ratio

**Supplemental Table 7.** One-year mortality in men with alcohol-related cirrhosis (n=16,940).

|  | **Number of deaths** | **Cumulative one-year mortality (%), (95% CI)§** | **Age-standardized one-year mortality rate per 1000 person-months, (95% CI)§** | **P-value for trend†** | **Unadjusted HR**  **(95% CI)§** | **Adjusted HR‡**  **(95% CI)§** |
| --- | --- | --- | --- | --- | --- | --- |
| **All-cause** |  |  |  | 0.514 |  |  |
| 1969-1979 | 1212 | 27.0 (25.7 to 28.3) | 27.6 (26.1 to 29.2) |  | 0.80 (0.73 to 0.87) | 0.89 (0.82 to 0.98) |
| 1980-1989 | 932 | 32.5 (30.8 to 34.3) | 35.6 (33.3 to 37.9) |  | Reference | Reference |
| 1990-1999 | 879 | 33.6 (31.8 to 35.4) | 38.0 (35.4 to 40.5) |  | 1.04 (0.95 to 1.14) | 0.95 (0.87 to 1.05) |
| 2000-2009 | 975 | 34.1 (32.3 to 35.8) | 37.7 (35.3 to 40.1) |  | 1.06 (0.97 to 1.16) | 0.86 (0.78 to 0.94) |
| 2010-2019 | 1307 | 31.8 (30.4 to 33.3) | 34.7 (32.8 to 36.6) |  | 0.98 (0.90 to 1.06) | 0.71 (0.65 to 0.77) |
| **Liver related** |  |  |  | 0.023 |  |  |
| 1969-1979 | 856 | 19.1 (18.0 to 20.3) | 19.4 (18.1 to 20.7) |  | 0.82 (0.74 to 0.91) | 0.92 (0.83 to 1.02) |
| 1980-1989 | 642 | 22.4 (20.9 to 24.0) | 24.3 (22.4 to 26.2) |  | Reference | Reference |
| 1990-1999 | 597 | 22.8 (21.2 to 24.4) | 25.7 (23.7 to 27.8) |  | 1.03 (0.92 to 1.15) | 0.94 (0.84 to 1.05) |
| 2000-2009 | 645 | 22.5 (21.0 to 24.1) | 24.9 (23.0 to 26.8) |  | 1.02 (0.91 to 1.13) | 0.84 (0.75 to 0.94) |
| 2010-2019 | 825 | 20.1 (18.9 to 21.3) | 21.8 (20.3 to 23.3) |  | 0.89 (0.81 to 0.99) | 0.71 (0.64 to 0.79) |
| **Non-liver related** |  |  |  | 0.039 |  |  |
| 1969-1979 | 356 | 7.9 (7.2 to 8.8) | 8.2 (7.3 to 9.1) |  | 0.75 (0.64 to 0.88) | 0.85 (0.73 to 1.00) |
| 1980-1989 | 290 | 10.1 (9.1 to 11.3) | 11.3 (10.0 to 12.6) |  | Reference | Reference |
| 1990-1999 | 282 | 10.8 (9.6 to 12.0) | 12.2 (10.8 to 13.7) |  | 1.08 (0.91 to 1.27) | 0.98 (0.83 to 1.16) |
| 2000-2009 | 330 | 11.5 (10.4 to 12.7) | 12.8 (11.4 to 14.2) |  | 1.15 (0.98 to 1.35) | 0.90 (0.76 to 1.05) |
| 2010-2019 | 482 | 11.7 (10.8 to 12.7) | 12.9 (11.7 to 14.0) |  | 1.16 (1.00 to 1.34) | 0.70 (0.60 to 0.82) |

† The decade 1980-1989 was used as reference.

‡ Adjusted for age, marital status, country of birth, decompensation, cardiovascular disease, diabetes, dementia, chronic obstructive pulmonary disease, cancer, and chronic kidney disease.

§ Abbreviations: CI, confidence interval; HR, hazard ratio

**Supplemental Table 8.** One-year mortality in women with alcohol-related cirrhosis (n=5718).

|  | **Number of deaths** | **Cumulative one-year mortality (%), (95% CI)§** | **Age-standardized one-year mortality rate per 1000 person-months, (95% CI)§** | **P-value for trend†** | **Unadjusted HR**  **(95% CI)§** | **Adjusted HR‡**  **(95% CI)§** |
| --- | --- | --- | --- | --- | --- | --- |
| **All-cause** |  |  |  | 0.316 |  |  |
| 1969-1979 | 249 | 20.9 (18.6 to 23.2) | 20.1 (17.6 to 22.6) |  | 0.83 (0.70 to 0.99) | 0.90 (0.75 to 1.07) |
| 1980-1989 | 232 | 24.3 (21.6 to 27.1) | 24.3 (21.2 to 27.5) |  | Reference | Reference |
| 1990-1999 | 256 | 28.5 (25.6 to 31.5) | 29.5 (25.9 to 33.1) |  | 1.20 (1.00 to 1.43) | 1.07 (0.90 to 1.28) |
| 2000-2009 | 283 | 26.5 (23.9 to 29.2) | 27.9 (24.6 to 31.1) |  | 1.11 (0.93 to 1.32) | 0.92 (0.77 to 1.10) |
| 2010-2019 | 431 | 26.9 (24.7 to 29.1) | 27.7 (25.1 to 30.4) |  | 1.13 (0.96 to 1.33) | 0.85 (0.72 to 1.02) |
| **Liver related** |  |  |  | 0.248 |  |  |
| 1969-1979 | 194 | 16.3 (14.2 to 18.4) | 15.6 (13.4 to 17.8) |  | 0.95 (0.77 to 1.18) | 1.03 (0.83 to 1.27) |
| 1980-1989 | 158 | 16.6 (14.3 to 19.0) | 16.5 (13.9 to 19.1) |  | Reference | Reference |
| 1990-1999 | 176 | 19.6 (17.1 to 22.2) | 20.2 (17.2 to 23.1) |  | 1.21 (0.97 to 1.50) | 1.10 (0.89 to 1.37) |
| 2000-2009 | 200 | 18.7 (16.5 to 21.1) | 19.7 (16.9 to 22.4) |  | 1.15 (0.93 to 1.42) | 0.98 (0.79 to 1.21) |
| 2010-2019 | 302 | 18.8 (17.0 to 20.8) | 19.4 (17.2 to 21.6) |  | 1.16 (0.96 to 1.41) | 0.94 (0.76 to 1.15) |
| **Non-liver related** |  |  |  | 0.944 |  |  |
| 1969-1979 | 55 | 4.6 (3.5 to 5.9) | 4.4 (3.3 to 5.6) |  | 0.58 (0.41 to 0.82) | 0.62 (0.43 to 0.87) |
| 1980-1989 | 74 | 7.8 (6.2 to 9.6) | 7.8 (6.0 to 9.6) |  | Reference | Reference |
| 1990-1999 | 80 | 8.9 (7.2 to 10.9) | 9.3 (7.3 to 11.4) |  | 1.18 (0.86 to 1.61) | 0.98 (0.71 to 1.35) |
| 2000-2009 | 83 | 7.8 (6.3 to 9.5) | 8.2 (6.4 to 10.0) |  | 1.02 (0.75 to 1.40) | 0.79 (0.57 to 1.09) |
| 2010-2019 | 129 | 8.0 (6.8 to 9.4) | 8.3 (6.9 to 9.8) |  | 1.06 (0.80 to 1.42) | 0.68 (0.49 to 0.93) |

† The decade 1980-1989 was used as reference.

‡ Adjusted for age, marital status, country of birth, decompensation, cardiovascular disease, diabetes, dementia, chronic obstructive pulmonary disease, cancer, and chronic kidney disease.

§ Abbreviations: CI, confidence interval; HR, hazard ratio

**Supplemental Table 9.** One-year mortality in patients with compensated alcohol-related cirrhosis (n=15,178).

|  | **Number of deaths** | **Cumulative one-year mortality (%), (95% CI)§** | **Age-standardized one-year mortality rate per 1000 person-months, (95% CI)§** | **P-value for trend†** | **Unadjusted HR**  **(95% CI)§** | **Adjusted HR‡**  **(95% CI)§** |
| --- | --- | --- | --- | --- | --- | --- |
| **All-cause** |  |  |  | 0.785 |  |  |
| 1969-1979 | 1160 | 23.4 (22.2-24.5) | 23.1 (21.7-24.4) |  | 0.78 (0.71-0.85) | 0.82 (0.75-0.90) |
| 1980-1989 | 840 | 28.7 (27.1-30.4) | 30.4 (28.4-32.5) |  | Reference | Reference |
| 1990-1999 | 698 | 30.5 (28.6-32.3) | 33.1 (30.6-35.5) |  | 1.08 (0.98-1.19) | 1.01 (0.92-1.12) |
| 2000-2009 | 615 | 29.7 (27.7-31.7) | 31.6 (29.1-34.1) |  | 1.04 (0.94-1.15) | 0.91 (0.82-1.01) |
| 2010-2019 | 834 | 28.6 (27.0-30.3) | 29.9 (27.9-32.0) |  | 1.00 (0.91-1.10) | 0.77 (0.69-0.85) |
| **Liver related** |  |  |  | 0.033 |  |  |
| 1969-1979 | 798 | 16.1 (15.1-17.1) | 15.8 (14.7-16.9) |  | 0.83 (0.74-0.92) | 0.84 (0.75-0.94) |
| 1980-1989 | 543 | 18.6 (17.2-20.0) | 19.5 (17.8-21.1) |  | Reference | Reference |
| 1990-1999 | 426 | 18.6 (17.0-20.2) | 20.1 (18.2-22.0) |  | 1.02 (0.90-1.16) | 0.99 (0.87-1.13) |
| 2000-2009 | 378 | 18.2 (16.6-19.9) | 19.4 (17.4-21.3) |  | 0.99 (0.87-1.13) | 0.97 (0.85-1.11) |
| 2010-2019 | 474 | 16.3 (14.9-17.6) | 17.0 (15.4-18.5) |  | 0.88 (0.77-0.99) | 0.83 (0.73-0.95) |
| **Non-liver related** |  |  |  | 0.026 |  |  |
| 1969-1979 | 362 | 7.3 (6.6-8.0) | 7.3 (6.5-8.0) |  | 0.69 (0.59-0.80) | 0.78 (0.67-0.91) |
| 1980-1989 | 297 | 10.2 (9.1-11.3) | 11.0 (9.7-12.2) |  | Reference | Reference |
| 1990-1999 | 272 | 11.9 (10.6-13.2) | 13.0 (11.4-14.5) |  | 1.19 (1.01-1.40) | 1.04 (0.89-1.23) |
| 2000-2009 | 237 | 11.4 (10.1-12.9) | 12.2 (10.7-13.8) |  | 1.13 (0.96-1.35) | 0.83 (0.69-0.98) |
| 2010-2019 | 360 | 12.4 (11.2-13.6) | 13.0 (11.6-14.3) |  | 1.22 (1.04-1.42) | 0.67 (0.57-0.79) |

† The decade 1980-1989 was used as reference.

‡ Adjusted for age, sex, marital status, country of birth, cardiovascular disease, diabetes, dementia, chronic obstructive pulmonary disease, cancer, and chronic kidney disease.

§ Abbreviations: CI, confidence interval; HR, hazard ratio

**Supplemental Table 10.** One-year mortality in patients with decompensated alcohol-related cirrhosis (n=7480).

|  | **Number of deaths** | **Cumulative one-year mortality (%), (95% CI)§** | **Age-standardized one-year mortality rate per 1000 person-months, (95% CI)§** | **P-value for trend†** | **Unadjusted HR**  **(95% CI)§** | **Adjusted HR‡**  **(95% CI)§** |
| --- | --- | --- | --- | --- | --- | --- |
| **All-cause** |  |  |  | 0.014 |  |  |
| 1969-1979 | 301 | 42.4 (38.7-46.0) | 51.1 (45.4-56.9) |  | 1.24 (1.06-1.45) | 1.32 (1.13-1.55) |
| 1980-1989 | 324 | 36.2 (33.1-39.4) | 40.5 (36.1-44.9) |  | Reference | Reference |
| 1990-1999 | 437 | 35.7 (33.0-38.4) | 40.6 (36.8-44.4) |  | 0.97 (0.84-1.12) | 0.93 (0.80-1.07) |
| 2000-2009 | 643 | 34.7 (32.5-36.8) | 38.9 (35.9-42.0) |  | 0.94 (0.83-1.08) | 0.84 (0.73-0.96) |
| 2010-2019 | 904 | 32.4 (30.6-34.1) | 35.8 (33.4-38.1) |  | 0.87 (0.77-0.99) | 0.72 (0.63-0.82) |
| **Liver related** |  |  |  | <0.001 |  |  |
| 1969-1979 | 252 | 35.5 (32.0-39.0) | 42.7 (37.4-47.9) |  | 1.31 (1.10-1.56) | 1.38 (1.16-1.64) |
| 1980-1989 | 257 | 28.8 (25.8-31.7) | 32.0 (28.1-36.0) |  | Reference | Reference |
| 1990-1999 | 347 | 28.3 (25.8-30.9) | 32.3 (28.9-35.7) |  | 0.97 (0.83-1.14) | 0.95 (0.81-1.12) |
| 2000-2009 | 467 | 25.2 (23.2-27.2) | 28.3 (25.7-30.9) |  | 0.86 (0.74-1.01) | 0.80 (0.68-0.93) |
| 2010-2019 | 653 | 23.4 (21.8-25.0) | 25.8 (23.8-27.7) |  | 0.80 (0.69-0.92) | 0.71 (0.61-0.82) |
| **Non-liver related** |  |  |  | 0.108 |  |  |
| 1969-1979 | 49 | 6.9 (5.2-8.9) | 8.5 (6.1-10.9) |  | 0.98 (0.68-1.42) | 1.08 (0.75-1.57) |
| 1980-1989 | 67 | 7.5 (5.9-9.3) | 8.5 (6.4-10.5) |  | Reference | Reference |
| 1990-1999 | 90 | 7.3 (6.0-8.9) | 8.3 (6.6-10.1) |  | 0.97 (0.71-1.33) | 0.85 (0.62-1.17) |
| 2000-2009 | 176 | 9.5 (8.2-10.9) | 10.7 (9.1-12.2) |  | 1.25 (0.94-1.65) | 0.98 (0.74-1.31) |
| 2010-2019 | 251 | 9.0 (8.0-10.1) | 10.0 (8.8-11.3) |  | 1.17 (0.89-1.53) | 0.78 (0.59-1.03) |

† The decade 1980-1989 was used as reference.

‡ Adjusted for age, sex, marital status, country of birth, cardiovascular disease, diabetes, dementia, chronic obstructive pulmonary disease, cancer, and chronic kidney disease.

§ Abbreviations: CI, confidence interval; HR, hazard ratio

**Supplemental Table 11.** Sensitivity analysis of one-year mortality in patients with compensated alcohol-related cirrhosis between 1987 and 2019 including non-bleeding oesophageal varices (n=8964).

|  | **Number of deaths** | **Unadjusted HR**  **(95% CI)‡** | **Adjusted HR†**  **(95% CI)‡** |
| --- | --- | --- | --- |
| **All-cause** |  |  |  |
| 1987-1989 | 232 | Reference | Reference |
| 1990-1999 | 737 | 1.07 (0.92 to 1.24) | 0.98 (0.85 to 1.14) |
| 2000-2009 | 701 | 1.03 (0.88 to 1.19) | 0.88 (0.76 to 1.03) |
| 2010-2019 | 960 | 0.96 (0.84 to 1.11) | 0.72 (0.62 to 0.84) |
| **Liver related** |  |  |  |
| 1987-1989 | 166 | Reference | Reference |
| 1990-1999 | 455 | 0.92 (0.77 to 1.01) | 0.88 (0.73 to 1.05) |
| 2000-2009 | 433 | 0.89 (0.74 to 1.06) | 0.83 (0.70 to 1.00) |
| 2010-2019 | 555 | 0.78 (0.65 to 0.93) | 0.69 (0.58 to 0.83) |
| **Non-liver related** |  |  |  |
| 1987-1989 | 66 | Reference | Reference |
| 1990-1999 | 282 | 1.43 (1.01 to 1.88) | 1.25 (0.96 to 1.64) |
| 2000-2009 | 268 | 1.38 (1.05 to 1.81) | 1.03 (0.79 to 1.36) |
| 2010-2019 | 405 | 1.43 (1.10 to 1.85) | 0.83 (0.64 to 1.09) |

† Adjusted for age, sex, marital status, country of birth, cardiovascular disease, diabetes, dementia, chronic obstructive pulmonary disease, cancer, and chronic kidney disease.

‡ Abbreviations: CI, confidence interval; HR, hazard ratio

**Supplemental Table 12.** Sensitivity analysis of one-year mortality in patients with decompensated alcohol-related cirrhosis between 1987 and 2019 excluding non-bleeding oesophageal varices (n=5268).

|  | **Number of deaths** | **Unadjusted HR**  **(95% CI)‡** | **Adjusted HR†**  **(95% CI)‡** |
| --- | --- | --- | --- |
| **All-cause** |  |  |  |
| 1987-1989 | 101 | Reference | Reference |
| 1990-1999 | 398 | 0.96 (0.77 to 1.19) | 0.91 (0.73 to 1.13) |
| 2000-2009 | 557 | 0.95 (0.77 to 1.17) | 0.83 (0.67 to 1.03) |
| 2010-2019 | 778 | 0.90 (0.73 to 1.11) | 0.74 (0.60 to 0.91) |
| **Liver related** |  |  |  |
| 1987-1989 | 87 | Reference | Reference |
| 1990-1999 | 318 | 0.89 (0.70 to 1.13) | 0.86 (0.68 to 1.10) |
| 2000-2009 | 412 | 0.81 (0.65 to 1.03) | 0.74 (0.59 to 0.94) |
| 2010-2019 | 572 | 0.77 (0.61 to 0.96) | 0.67 (0.53 to 0.84) |
| **Non-liver related** |  |  |  |
| 1987-1989 | 14 | Reference | Reference |
| 1990-1999 | 80 | 1.39 (0.79 to 2.45) | 1.20 (0.68 to 2.13) |
| 2000-2009 | 145 | 1.78 (1.03 to 3.08) | 1.36 (0.78 to 2.36) |
| 2010-2019 | 206 | 1.72 (1.00 to 2.95) | 1.13 (0.65 to 1.95) |

† Adjusted for age, sex, marital status, country of birth, cardiovascular disease, diabetes, dementia, chronic obstructive pulmonary disease, cancer, and chronic kidney disease.

‡ Abbreviations: CI, confidence interval; HR, hazard ratio

**Supplemental Table 13.** One-year first-time rehospitalization rates in patients with alcohol-related cirrhosis (n=22,658).

|  | **Number of events** | **Cumulative one-year incidence (%), (95% CI)§** | **Age-standardized one-year rehospitalization rate per 1000 person-months, (95% CI)§** | **P-value for trend†** | **Unadjusted HR**  **(95% CI)§** | **Adjusted HR‡**  **(95% CI)§** |
| --- | --- | --- | --- | --- | --- | --- |
| **All-cause** |  |  |  | 0.004 |  |  |
| 1969-1979 | 3679 | 64.8 | 110.3 (106.7-113.9) |  | 0.75 (0.71-0.79) | 0.77 (0.74-0.81) |
| 1980-1989 | 2851 | 74.7 | 158.2 (152.4-164.0) |  | Reference | Reference |
| 1990-1999 | 2582 | 73.4 | 163.3 (157.0-169.6) |  | 1.03 (0.98-1.09) | 1.00 (0.94-1.05) |
| 2000-2009 | 2932 | 74.6 | 170.5 (164.3-176.6) |  | 1.08 (1.02-1.13) | 0.99 (0.94-1.04) |
| 2010-2019 | 4212 | 73.8 | 165.7 (160.7-170.8) |  | 1.07 (1.02-1.12) | 0.96 (0.91-1.01) |

† The decade 1980-1989 was used as reference.

‡ Adjusted for age, marital status, country of birth, decompensation, cardiovascular disease, diabetes, dementia, chronic obstructive pulmonary disease, cancer, and chronic kidney disease.

§ Abbreviations: CI, confidence interval; HR, hazard ratio


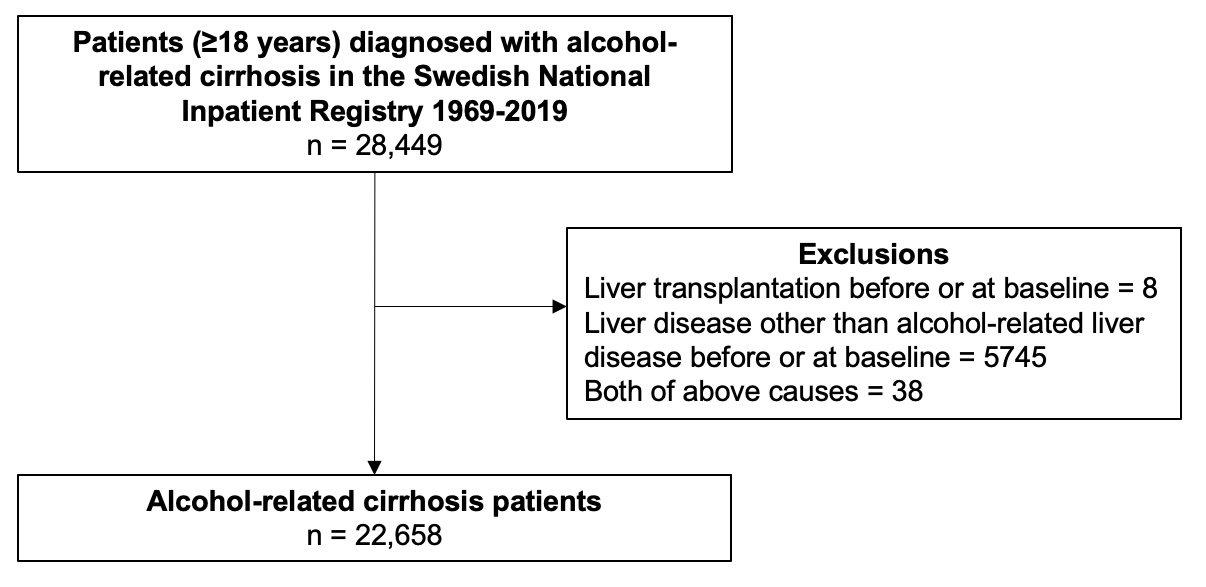


**Supplemental Figure 1.** Flowchart of the study population.

**
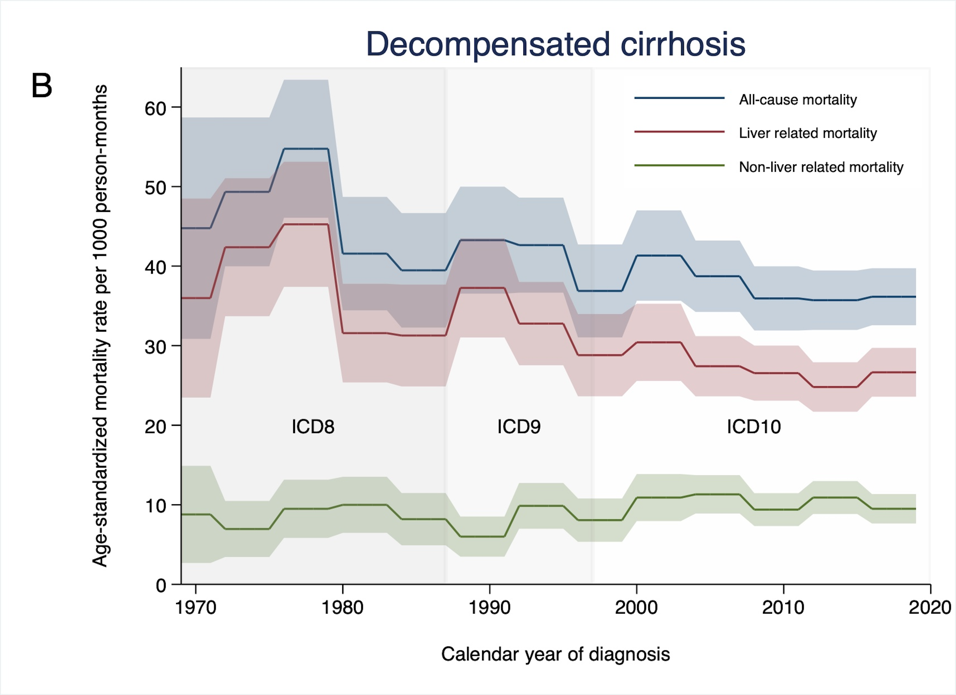

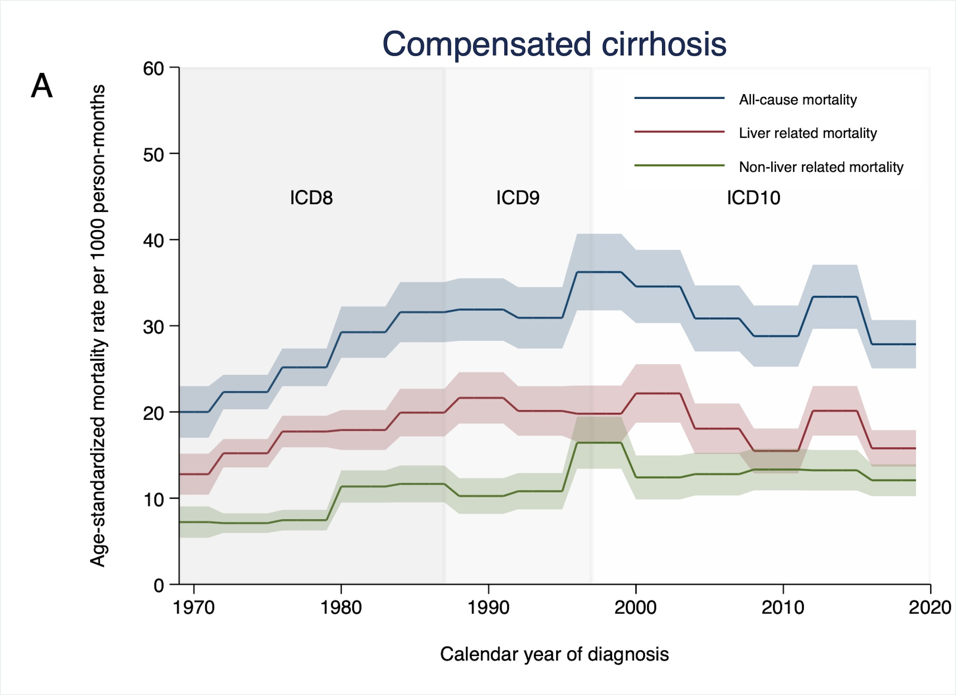
**

**Supplemental Figure 2.** Time trends in mortality per 1000 person-months during the first year after a diagnosis of alcohol-related cirrhosis in Sweden between 1969 and 2019 for patients with compensated cirrhosis (A), and decompensated cirrhosis (B). The shaded areas represent the 95% confidence intervals.
